# Transmission of the gut microbiome in cohousing goats and pigs

**DOI:** 10.3389/fmicb.2022.948617

**Published:** 2022-09-07

**Authors:** Tingting Zhang, Mao Li, Tao Shi, Yueyang Yan, Zhannur Niyazbekova, Xihong Wang, Zongjun Li, Yu Jiang

**Affiliations:** ^1^College of Animal Science and Technology, Northwest A&F University, Yangling, Shaanxi, China; ^2^Tropical Crops Genetic Resources Institute, Chinese Academy of Tropical Agricultural Sciences, Haikou, Hainan, China; ^3^College of Veterinary Medicine, Jilin University, Changchun, Jilin, China

**Keywords:** social interaction, goat, pig, gut microbiome transmission, metagenome-assembled genomes

## Abstract

Social interaction facilitates the horizontal transmission of the microbiota between different individuals. However, little is known about the level of microbiota transmission in different livestock animals and different digestive tracts. The Hainan black goat and Wuzhishan pig are typical tropical local breeds on Hainan Island in China. Thus, we sampled and analyzed the gut microbiome in Hainan black goats (cecum and rumen) and Wuzhishan pigs (cecum) to study horizontal transmission by rearing them in the same pen (six goats and six pigs) or separate pens (nine goats and nine pigs). *De novo* assembly and binning recovered 3,262 strain-level and 2,488 species-level metagenome-assembled genomes (MAGs) using ∼1.3 Tb sequencing data. Of these MAGs, 1,856 MAGs were identified as novel strain. Compared with goats living in separate pens, social interaction in the same pen promotes community homogeneity in the rumen microbiome (*P* < 0.05) and the cecum microbiome (*P* < 0.05), respectively. Notably, approximately 7.08% (231/3262) of the gut microbial population could transmit during cohousing, 12 strains only in inter-species transmission, versus 190 strains only in intra-species transmission, and 10 strains only in foregut and hindgut transmission. In addition, the social contact group has high transmitted strain abundance, which is correlated with community composition. This study provided a new insight into the influence of social interaction on the animal gut microbiota.

## Introduction

The digestive system harbors a diverse and complex microbiota which collectively modulates host health, development, and physiology (Wang et al., 2018; Guo et al., 2020). Colonization of the gut microbiota is a complex process that is affected by both host genetics (Zhou et al., 2018) and environmental factors (Eisenstein, 2020). In certain circumstances, the influence of environmental factors appears to outweigh host genetics in shaping microbiome composition (Henderson et al., 2015; Ji et al., 2018). Several recent studies in both humans and non-human primates provide strong evidence for the contribution of social interaction to microbiome assembly (Song et al., 2013; Tung et al., 2015; Li et al., 2016; Grieneisen et al., 2017; Sharma et al., 2019). Song et al. (2013) found that household members shared more microbiota than individuals from different households, thereby decreasing inter-individual variation. Tung et al. (2015) showed that social interaction could predict microbiome structure in wild baboons. In addition, a study examined gut microbiome composition in nine sympatric wild non-human primate species and found that microbiota varied with host species but, importantly, also by social groups within species (Gogarten et al., 2018). Despite the social interaction playing an important role in gut microbiome composition, few studies focused on non-primate animals.

Use of the 16S rRNA gene analyses does not have sufficient taxonomic and functional resolution, while metagenomic shotgun sequencing has the potential to reveal microbial heterogeneity at the strain-level, which may be crucial to identifying and assessing the transmission of strains with distinct genetic repertoires (Nayfach et al., 2016; Yassour et al., 2018). Compared with bumblebees, honeybees often possess a higher level of microbial strain variation, which also exhibited more complex gene repertoires linked to polysaccharide metabolism (Su et al., 2021). In addition, the newly emerged worker bees share more strains with their queens, suggesting a vertical transmission of strains from queens to the newborn workers (Su et al., 2021). Yassour et al. (2018) found two patterns of mother-to-child microbial transmission at strain-level. A *Bacteroides uniformis* strain lacking utilization gene cluster in the mother’s strain was inherited to the child, implying that the monther’s strain has a selective advantage in the infant gut (Yassour et al., 2018). Therefore, strain-level metagenomic profiling is needed to infer the transmission at higher taxonomic resolutions.

Hainan Island, in the South China Sea, has a tropical climate that is hot and humid, prompting the local animals to have differences from intensive commercial species in physical and life modes (Zhu, 2016). Hainan black goats and Wuzhishan pigs are native breeds that have long been bred in the unique natural ecological environment of Hainan Island (Jiang et al., 2020; Shao et al., 2021). In addition, the Wuzhishan pig is a famous miniature pig breed and is regarded as an ideal experimental animal model (Shao et al., 2021). Hainan black goats as ruminants have tender and delicious meat, making them a popular choice among consumers (do Rosario et al., 2016). Thus, to detect the microbiome transmission during cohousing, we chose two kinds of animals with different feeding habits and selected Hainan black goat to analyze the transmitted strain between the foregut and hindgut.

Using metagenome-assembled genomes (MAGs), we examined changes in the gut microbiome community and microbial transmission from cohousing goats and pigs. Our results provide insights into the gut microbiota during social interaction and expand our understanding of microbiome transmission. Among them, 1,856 new strain-level MAGs broaden the useful microbial resources for the tropical climate system.

## Materials and methods

### Study design, sample collection, and sequencing

In this study, 15 newborn Hainan black goats and 15 newborn Wuzhishan pigs were used, and they weaned at 3 and 1.5 months, respectively. After weaning, six goats and six pigs were cohoused in a new barn, while left goats and pigs still lived in the separate barns. Rumen and cecum samples were collected at weaning (0 day), 3 and 12 months after co-housing, respectively (Supplementary Figure 1). Three goats and pigs from each group were slaughtered 3 h later after the morning feeding at sample collecting day. All fresh samples were frozen in liquid nitrogen before being transferred to the laboratory in a dry-ice pack and promptly kept at −80°C before total DNA extraction. DNA extractions followed the instructions using the DNeasy PowerLyzer PowerSoil Kit (Qiagen, United Kingdom). Shotgun sequencing was performed on an Illumina NovaSeq platform.

### Metagenome assembly and annotation

Illumina sequencing data adaptors were trimmed using Trimmomatic (Bolger et al., 2014) and the subsequent trimmed reads were aligned to the reference genome to remove host-derived DNA contamination. The clean reads per sample were performed using MegaHit (Li et al., 2015), which is included in MetaWRAP (Uritskiy et al., 2018). The contigs were retained for the subsequent analysis with the length ≥ 1 kb. Metagenomic binning was applied to recover individual genomes using MateBAT2, Maxbin2 and CONCOCT in the binning module. Three sets of draft bins were further analyzed using the bin_refinement module of the MetaWRAP pipeline with options -c 50 -x 10. The bins ≥ 50% complete were re-assembled using the SPAdes. To determine whether the same bins have been reconstructed *via* different samples, all bins were dereplicated using dRep at a threshold of 99% average nucleotide identity (ANI). The CheckM estimate results of MAGs meeting the following thresholds were retained: Genome Quality ≥ 50; contamination ≤ 10%; and completeness ≥ 50% [genome Quality = Completeness−5 × Contamination + log(N50)].

ORFs were predicted by Prodigal (v2.6) based on the default parameters and the function assignment was performed using the Prokka software (Seemann, 2014). The 16S RNA genes in MAGs sequences were predicted using Barrnap (v 0.9). Then, the MAGs-derived proteins were searched against the carbohydrate-active (CAZy^1^) database using dbCAN2 (Zhang et al., 2018) and the Kyoto Encyclopedia of Genes and Genomes (KEGG) database (Kanehisa et al., 2017) using KofamKOALA (Aramaki et al., 2020). Reads from each sample were aligned against the contigs of MAGs by the Salmon software (Patro et al., 2017). The abundance of each contig was calculated in contigs per million (CPM), and then the median CPM of the contig was used as the abundance of MAGs.

### Taxonomic classification, phylogenetic trees, and species-level clustering of metagenome-assembled genomes

The Genome Taxonomy Database Toolkit (GTDB-Tk^2^) pipeline was used for the taxonomic classification of the 3,262 MAGs (Parks et al., 2018). In short, the GTDB-Tk classified the bins based on ANI to the reference genome, placement in the bacterial or archaeal reference genome tree, and relative evolutionary distance. MAGs were clustered into species-level genome bins at the threshold of 95% ANI. Phylogenetic relationships among the 2,488 species-level genome bins were computed by the total branch length based on proteins. The phylogenetic tree was produced from concatenated protein sequences using PhyloPhlAn and re-rooted manually using iTOL at the branch between archaea and bacteria. The tree was used to calculate the phylogenetic gain at different taxonomic levels using the pd_clade routine in GenomeTreeTk (v0.1.6).^3^ We downloaded 48,622 microbial genomes from GTDB (31,910 genomes) (Parks et al., 2018), ruminant gastrointestinal tract (10,737 genomes) (Xie et al., 2021), and pig gut (6,339 genomes) (Chen et al., 2021). All these genomes were used as reference genomic datasets for the identification of novel microbial genomes from 3,262 MAGs using FastANI (Jain et al., 2018). Genomes were determined as novel strains based on < 99% ANI and defined as novel species based on < 95% ANI. All the sequenced reads from our study were mapped to two datasets using Kraken2 to estimate the read classification. Two combined databases: a common database consisting of the bacterial, archaeal, fungal, and protozoan genomes in GenBank, and the GenBank database plus the MAGs.

### Strains filtering, identification of microbial single nucleotide variants and detection of microbial transmission

To determine the presence or absence of strains in samples, the strains were filtered to satisfy the following criteria: horizontal coverage (breadth) of ≥ 10%, average vertical coverage (depth) ≥ 0.15 x (Schmidt et al., 2019). Microbial Single Nucleotide Variants (SNVs) were called using metaSNV software (Costea et al., 2017). In short, the transmission score (S_T_) quantifies how much the similarity between two sample SNV profiles from social contact group, and between foregut and hindgut SNV profiles within an individual. Each potential SNV requires support by at least two reads at a base call quality of Phred ≥ 15. To identify the microbial transmission event, we applied a text mining approach (Schmidt et al., 2019) to calculate the S_T_. Next, we calculated the incidence of each allele across screening samples. For these, we calculated the probabilities in paired samples observations for each of the four possible cases: any given allele i could either be present in both samples (p_1_,_1_), absent in both samples (p_0_,_0_), or present in one but absent in the other sample (p_1_,_0_ and p_0_,_1_):


p1,1⁢(i)=fpaired⁢sample⁢ 1⁢(i)-fpaired⁢sample⁢ 2⁢(i)

p0,0⁢(i)=(1-fpaired⁢sample⁢ 1⁢(i))-(1-fpaired⁢sample⁢ 2⁢(i))

p1,0⁢(i)=fpaired⁢sample⁢ 1⁢(i)-(1-fpaired⁢sample⁢ 2⁢(i))

p0,1⁢(i)=(1-fpaired⁢sample⁢ 1⁢(i))-fpaired⁢sample⁢ 2⁢(i)

For each pair of aligned samples, we calculated the raw and logarithmic probability of SNV profile overlap (*L*_*obs*_) observed, which quantifies how likely the observed average allele profile agreement between two samples.


$$L_{o\,b\,s}=\left(\sum_{i}^{1,1}l\,o\,g\,\left(p_{1,1}\,\left(i\right)\right)+\sum_{j}^{0,0}l\,o\,g\,\left(p_{0,0}\,\left(j\right)\right)\right)-\left(\sum_{k}^{1,0}l\,o\,g\,\left(p_{1,0}\,\left(k\right)\right)+\sum_{l}^{0,1}l\,o\,g\,\left(p_{0,1}\,\left(l\right)\right)\right)$$

Similarly, we computed the log-likelihood of the least likely agreement case (*L*_*min*_) per allele:


$$L_{m\,i\,n}=\sum_{i}m\,i\,n\,\left(l\,o\,g\,\left(p_{1,1}\,\left(i\right)\right),l\,o\,g\,\left(p_{0,0}\,\left(i\right)\right)\right)$$

Next, a raw probability score (*P*_*raw*_) for the observed allele agreement between a given paired samples is calculated: *P*_*raw*_ = *L*_*obs*_/*L*_*min*_. We defined the S_*T*_(t, s) for starin t in subject s as a standard Z score of the *P*_*raw*_: *S*_*T*_ = (*P*_*raw*_(*s*)−μ_*raw*_)/σ_*raw*_.

Microbial transmission events that were identified in pair of samples from social contact group and the foregut-hindgut pair of samples from the same individual have been divided into three types: intra-species transmission events, in which goats or pigs spread microbiota within their own species; inter-species transmission events, in which microbiota transmit between goats and pigs; and foregut-hindgut transmission events, in which microbiota spread between the foregut and hindgut within an individual.

### Statistical analysis

Permutational multivariate analysis of variance (PERMANOVA) quantifies the variation among the samples that partitions social contact group and control group distances to permit the assessment of the effect of an exposure or intervention (grouping factor) on the sampled microbiome (Kelly et al., 2015). PERMANOVA was performed using the adonis2 function from the Vegan package with default settings with 999 unrestricted permutations and the Monte Carlo *P* value was calculated. *P* < 0.05 was considered statistically significant. Spearman correlation analysis was used to assess relationships between stain richness and community composition and transmission strain abundance in the control group or social contact group (goat rumen, goat cecum, and pig cecum).

## Result

### Reconstructing metagenome-assembled genomes from the gastrointestinal tract microbiota

**FIGURE 1:**
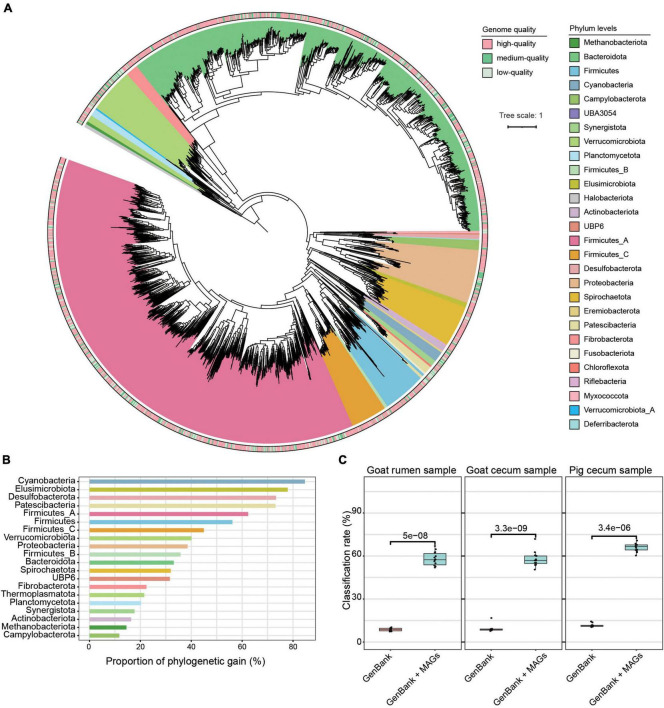
Taxonomic annotation and phylogenetic tree of 3,262 metagenome-assembled genomes (MAGs). **(A)** The maximum-likelihood tree of the 3,262 MAGs identified in this study was produced from concatenated protein sequences using PhyloPhlAn. **(B)** The phylogenetic gain contributed by the microbial tree of the gut provided by the unknown species-level genome bins is shown as proportional increases in branch length per phylum (left) and absolute branch lengths (right). **(C)** Comparison of the read classification rates of all the digesta samples using the following datasets: a common database consisting of all complete microbial genomes in RefSeq and the GenBank database plus MAGs. The Wilcoxon rank-sum test was used to assess the differences.

Using 1.3 Tb metagenomic sequencing data, we created a non-redundant set of 3,262 strain-level MAGs (< 99% ANI) and 2,488 species-level MAGs (< 95% ANI) with genome quality score ≥ 50, ≥ 50% completeness, and ≤ 10% contamination (Supplementary Figure 2 and Supplementary Table 1). Among these MAGs, 1,152 were estimated as high-quality with completeness ≥ 90% and contamination ≤ 5%, 1,497 were medium-quality (completeness ≥ 70% and contamination ≤ 10%), and 613 were low-quality (completeness ≥ 50% and contamination ≤ 5%). As shown in Figure 1A and Supplementary Table 1, the dominant phyla according to the number of MAGs that were assigned to Firmicutes_A (*n* = 1,246), Bacteroidota (*n* = 1,194) (dominated by classes Clostridia and Bacteroidia), followed by Proteobacteria (*n* = 136), Firmicutes (*n* = 120), Verrucomicrobiota (*n* = 117), and Spirochaetota (*n* = 106). The dominant orders included Bacteroidales (*n* = 1,189), Oscillospirales (*n* = 680), Lachnospirales (*n* = 282), and Christensenellales (*n* = 170), while the dominant families were Bacteroidaceae (*n* = 507), Lachnospiraceae (*n* = 274), Acutalibacteraceae (*n* = 231), UBA932 (*n* = 229), Oscillospiraceae (*n* = 160), CAG-272 (*n* = 142), and Ruminococcaceae (*n* = 111). At the genus level, the dominant groups included Prevotella and RC9. Thirty-four archaeal MAGs belonging to three phyla, Thermoplasmatota (*n* = 18), Methanobacteriota (*n* = 9), and Halobacterota (*n* = 7) were identified. The read classification rate was increased by using these genomes, and we observed over 50% reads classification rate across this study metagenomic datasets (Figure 1C). Furthermore, across published metagenomic datasets, these MAGs showed a read classification rate of more than 30% (Supplementary Figure 3).

Using species-level thresholds (≥ 95% ANI and ≥ 65% alignment), 1,856 of the MAGs did not match any available databases (Supplementary Table 1). Of these, 663 were high-quality genomes, 835 were medium-quality genomes, and 358 were low-quality genomes. The 1,856 MAGs were assigned to 27 phyla, 70 orders, 131 families, and 336 genera, with 17.6% of these MAGs unable to be assigned to a recognized genus, implying that a significant fraction of the MAGs are likely novel genera. Prevalent among these MAGs classified at the order level were Oscillospirales (28.0%), Bacteroidales (27.9%), and Lachnospirales (10.5%), while the top genera all belonged to the order Bacteroidales, including Prevotella (5.6%) and RC9 (4.8%). In addition, to understand the phylogenetic position of the uncultured strains, we placed the 3,262 MAGs in a maximum-likelihood tree and found that the unknown MAGs improved phylogenetic diversity by an average of 49.8 and 11.3% for bacterial and archaeal lineages, respectively (Figure 1B).

### Functional characteristics of metagenome-assembled genomes in different gastrointestinal tracts

We explored the proteomic contents of our datasets and their putative functions. A total of proteins that could be annotated according to one or both approaches were 2,753,800, of which 2,589,938 and 295,216 proteins were annotated by using the KEGG database and CAZy, respectively (Supplementary Tables 2, 3).

To investigate the ability of the identified microbial to produce short-chain fatty acids (SCFAs), we explored the genes that are required to produce some components such as acetate, propionate, and butyrate (Supplementary Table 4). As a result, genes required for the complete acetate-producing pathway were found in 1,248 MAGs, which were generally assigned in the Firmicutes_A and Bacteroidota phyla. Complete enzymes for the propionate production (*via* propionate pathway by the conversion of L-Lactate, succinate, and acetyl-CoA to propanoyl-CoA) were found in 239 MAGs of the Bacteroidota and Firmicutes_A. Enzymes encoded by genes for butyryl-CoA dehydrogenase, methylmalonyl-CoA mutase, methylmalonyl-CoA epimerase, enoyl-CoA hydratase, formate C-acetyltransferase, pyruvate ferredoxin oxidoreductase alpha subunit, acetate kinase, and phosphate acetyltransferase were common in these genomes. Sixty MAGs were identified to form butyrate (from acetyl-CoA through the typical pathway *via* butyrate kinase or acetate CoA-transferase).

Carbohydrate active enzymes (CAZymes) are enzymes involved in the metabolism and binding of carbohydrates. The majority of classes belonged to glycoside hydrolases (GHs), with 141,404 genes identified. The next glycosyltransferases (GTs; 74,345) and carbohydrate esterases (CEs; 50,099) were the most annotated groups (Supplementary Table 5). And then, the remaining three categories including carbohydrate-binding modules (CBMs; 47,009), polysaccharide lyases (PLs; 15,498), and auxiliary activities (AAs; 13,240), contained fewer proteins. Bacteroidota, Firmicutes_A, and Verrucomicrobiota showed high proportions of GHs, while Bacteroidota and Firmicutes_A showed high proportions of GTs. Notably, the most commonly identified GH was the GH109 family, which combines many enzymes responsible for the degradation of glycoproteins. Furthermore, is followed by GH2 and GH3 families, which are mostly related to cellulose and hemicellulose degradation.

### Gastrointestinal tract microbiota changes correlate with social interaction

**FIGURE 2:**
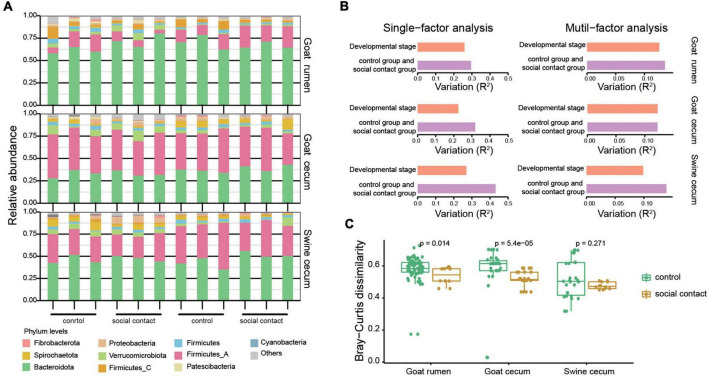
Change in the gut microbiota following environmental conversion. **(A)** Relative abundance of microbial phyla for the goat rumen, goat cecum, and pig cecum samples. **(B)** A bar plot summarizing the results of single and multiple factors permutational multivariate analysis of variance (PERMANOVA) results in the goat rumen, goat cecum, and pig cecum samples. The variations were derived from the between-sample Bray-Curtis distance. **(C)** Stability of strain-level composition profiles between control and social contact group as measured by Bray-Curtis dissimilarity. The Wilcoxon rank-sum test was used to assess the differences.

As expected, each individual had a distinct microbial composition at a coarse phylum level (Figure 2A). Cecum microbial communities were dominated by strains from the Bacteroidetes (34.9% in goat, 47.7% in pig), Firmicutes_A (46.4% in goat, 33.3% in pig), Verrucomicrobiotes (4.1% in goat, 3.1% in pig), Firmicutes (3.5% in goat, 3.3% in pig), and Spirochaetota (3.0% in goat, 4.3% in pig) phyla. Meanwhile, Bacteroidetes (65.9%) and Firmicutes_A (15.8%) were the two prevalent phyla in the rumen communities. Interestingly, rumen Bacteroidota abundance was higher than the cecum, although Firmicutes_all showed the opposite pattern (Supplementary Figure 4). Consequently, the Firmicutes to Bacteroidota ratio was lowest in the rumen and highest in the cecum of goat (Supplementary Figure 4).

Using permutational multivariate analysis of variance, we investigated the effects of host and environmental variables on the microbiota. The environmental factor exerted the main effects in both the single-and multi-factor analyses, followed by the developmental stage (Figure 2B). Since the sampling strategy applied in this study was optimized to compare microbial compositions across the control and social contact groups, we focused on the comparative analysis between the control group and the social contact group and left the comparisons of other factors for future studies. Moreover, we analyzed the relative abundances of the MAGs in the control group and social contact group, and observed no significant differences in alpha diversities between the two groups in goat rumen, goat cecum, and pig cecum, as evaluated by the Shannon and richness indexes (Supplementary Figure 4). The social contact microbial communities exhibit significantly similar features in goat rumen and goat cecum (*P* = 0.014, *P* = 5.4e–05, Figure 2C). However, the social contact microbial communities of the pig cecum were similar but not significant (Figure 2C). Overall, these results indicate that microbiota may be transmitted during the microbiota in co-housed animals.

### Transmission of microbes during the social interaction

**FIGURE 3:**
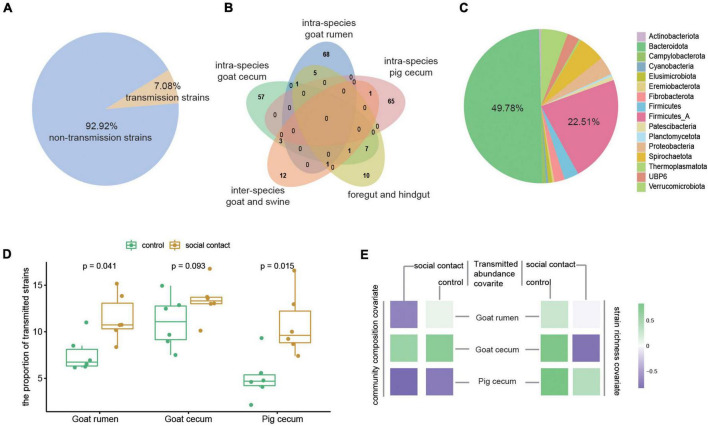
Microbiome in different transmission events. **(A)** The transmission microbiota’s proportion of the total number of strains. **(B)** Overlap in the strains of the different transmission events (right). **(C)** The phylum annotation of the transmission microbiota proportion. **(D)** The proportion of transmission microbiota in total microbiome abundance in the control and social contact groups from goat rumen, goat cecum, and pig cecum. The Wilcoxon rank-sum test was used to assess the differences. **(E)** Tests for the relationship between the abundance of transmission strains in the control or social contact groups (goat rumen, goat cecum, and pig cecum) and stain richness (ρ_Spearman_) and community composition (ρ_Spearman_).

We further investigated the transmission of strain-level MAGs following cohousing by comparing different species of animals that cohabited. In addition, we also determined the transmission strains in the foregut and hindgut in the same sample. The 7.08% of the microbiota (231 out of a total of 3,262) were detected as transmission microbiota (Figure 3A). The majority of these strains were only assigned to within-species microbial transmission (190, 82.25% out of 231), after which the between-species group (12, 5.19%) was the most assigned group, and the foregut-hindgut transmission events (10, 4.33%) contained fewer strains (Figure 3B). The transmitted strains were assigned to 16 phyla, and the dominant phyla were Bacteroidota (49.78%) and Firmicutes_A (22.51%) (Figure 3C). Transmissible strains accounted for a high fraction of classifiable microbial abundance in the social contact group (Figure 3D). The abundance of transmission microbiome in the social contact group was found to negatively correlate with community composition in goat rumen (ρ_Spearman_ = −0.65). Goat cecum transmitted strains abundance correlated with strain richness (social contact group: ρ_Spearman_ = −0.77; control group: ρ_Spearman_ = 0.77), and with community composition (social contact group: ρ_Spearman_ = 0.54; control group: ρ_Spearman_ = 0.65). Pig cecum transmitted strains abundance (control group) correlated with strain richness (ρ_Spearman_ = 0.82) and community composition (ρ_Spearman_ = −0.71) (Figure 3E).

## Discussion

Social interaction, in an enriched social environment, plays an important role in an individual’s health (Spor et al., 2011; Iida et al., 2013). One mechanism for this effect is the interaction of animal-associated microbiota (Rothschild et al., 2018). This study on the effects of cohabitation with different species on the gut microbiome in non-primates. From our dataset, we constructed 3,262 MAGs and 1,856 novel MAGs, considerably extending prior MAGs (Parks et al., 2018; Chen et al., 2021; Xie et al., 2021). The dataset increased the reads classification rate by over 30% and provides the possibility for analysis of microorganism transmission. The gut microbiome similarity within the social contact group increased during cohabitation. Moreover, we found that within-species transmission is the main type in the gut microbiome.

Our results highlight the importance of cohousing mediated transmission in shaping gut microbiomes. Previous studies have reported that social interaction can lead to a similarity in the structures of the gut microbiome (Tung et al., 2015; Zhu et al., 2020, 2021). Although these studies have great ecological validity, it is difficult to understand the effects of social interactions on microbiome similarity from the combined effects of shared dietary intake or exposure to local environmental microbes. Animals in our study were fed identical diets in each species. Our findings indicated that cohabitation with diverse species had an effect on the gut microbiome community by increasing gut microbiota similarity. Despite the small number of transmitted trains in the gut microbiome (goat rumen, goat cecum, and pig cecum), we discovered that transmitted strains were frequent throughout microbial phyla. Previous research reported that humans have a greater gut microbiome among households and between couples than non-related people, and there was no consistent indication of transmission across any specific phyla (Brito et al., 2019). All these results showed that transmission may be largely driven by chance events and indirect transfer. Although we could not determine whether or not direct transmission occurred and the direction of transmission, our results showed that the number of intra-species transmission strains was higher than that of inter-species transmission strains, which may be correlated with diet (Zhu et al., 2021). Based on the abundance analysis of the putatively transmitted gut microbiome, we observed that the social contact group has a high proportion of transmitted strains, and correlates with the microbial community. One possible reason for this pattern is that when microbiota colonizes a new host, they become more abundant to occupy the available gut microbial niches (Tung et al., 2015).

Using culture-independent approaches to recover the genomes, which have become commonly used for species discovery and characterization (Almeida et al., 2019). In this study, we constructed 1,856 novel strains from our dataset. Among all the MAGs, the novel bacterial taxa derived from the *Clostridia* class were more abundant, which were mainly distributed in the ten families such as Lachnospiraceae, Acutalibacteraceae, Oscillospiraceae, CAG-272, Ruminococcaceae, Borkfalkiaceae, CAG-74, Saccharofermentanaceae, and CAG-138. Many commensal *Clostridia* species belong to a large group of obligate anaerobic and highly diverse bacteria, which are thought to be responsible for the maintenance of gut homeostasis. The high diversity of unclassified genomes affiliated with *Clostridia* has also been reported in the cecum microbiome (Glendinning et al., 2020) and the bovine rumen microbiome (Stewart et al., 2019). Phylogenetic analysis revealed that unknown genomes expand the diversity of bacterial and archaeal lineages. In addition, we provided a dataset of gut microbial genomes, which not only increases the reads classification but also enables us to analyze the transmission of microorganisms.

The Short-chain fatty acids (SCFAs), mainly acetate, propionate, and butyrate, are the principal products of the microbial fermentative activity in the gastrointestinal tract and have significant effects on intestinal health and immunity (Koh et al., 2016; Baaske et al., 2020). Propionate that is mainly transported to the liver is an important energy source for the host (Koh et al., 2016). The majority of bacteria belong to the Lachnospiraceae family, carrying complete genes for propionate production. Moreover, butyrate is an important energy source for the ruminal epithelium enterocytes and is involved in the maintenance of gut homeostasis (Koh et al., 2016; McNabney and Henagan, 2017; Baaske et al., 2020). In this study, the majority of taxa encoding enzymes required for butyrate production were Bacteroidota and Firmicutes_A. Notably, the Bacteroidaceae and Lachnospiraceae families carrying genes from the butyrate pathway have the metabolic capability to degrade and utilize plant-derived fibers as nutrients (Vital et al., 2014; Onrust et al., 2015; Segura-Wang et al., 2021). Furthermore, all genes required for reductive acetate in the Lachnospiraceae family, have also been reported in other studies (Bhattacharya et al., 2015). Therefore, the identification of strains carrying the enzymes necessary for acetate, propionate, and butyrate production, is an important step toward future modulation of the gut microbiota to improve the growth performance of animals.

The gastrointestinal tract microbiome carries a large repertoire of genes encoding enzymes that can breakdown polysaccharides (Lillington et al., 2020; Wardman et al., 2022). In the current study, GHs were discovered in the gut microbiota of a variety of species that can catalyze the hydrolysis of glycosidic bonds found in complex carbohydrates (Bohra et al., 2019). The high abundance observed for the GH109 family underlines its importance, which could cleave the terminal N-acetylgalactosamine from the A antigen (Wardman et al., 2022). Furthermore, the GH2 and GH3 families contain widely distributed enzymes with a range of degradative activity on plant cell wall polysaccharides (Dai et al., 2015; Chang et al., 2018). This provides a basis for more effort to explore the gastrointestinal tract microbiome, and we will be able to identify a large number of enzymes of industrial value.

## Conclusion

This report offers novel insight into the relevant microbial transmission patterns within cohousing goats and pigs. It suggests that within-species microbial transmission is the main transmission pattern. Transmission of potential microbiome communities is relevant to the overall microbiome composition of individuals, and thus social interaction-derived changes in microbiomes may potentially impact the health status of individuals. In addition, a total of 3,262 strain-level MAGs, 2,488 species-level MAGs, and 1,856 unknown strain-level MAGs from our dataset were reconstructed and detected from the gut microbiome.

## Data availability statement

The datasets presented in this study can be found in online repositories. The names of the repository/repositories and accession number(s) can be found below: NCBI SRA BioProject, accession no. PRJNA792486.

## Ethics statement

The animal study was reviewed and approved by Northwest A&F University. Written informed consent was obtained from the owners for the participation of their animals in this study.

## Author contributions

TZ, ZL, XW, and YJ designed the research project. TZ, TS, and YY analyzed the data. TZ and ML collected animal samples. TZ wrote the manuscript with significant input from all authors. TZ, ZL, ZN, and YJ edited the language of the manuscript. All authors contributed to the article and approved the submitted version.

## Footnotes

1
http://www.cazy.org

2
http://gtdb.ecogenomic.org

3
https://github.com/dparks1134/GenomeTreeTk

## Supplementary material

Supplementary Figure 1 Schematic representation of the study design. The experimental design was used to characterize the microbiota composition of the social contact group (co-housed animals; *n* = 15 Hainan black goats and *n* = 15 Wuzhishan pigs) and the control group (non-cohoused; *n* = 15 Hainan black goats and *n* = 15 Wuzhishan pigs) over time. Hainan black goats were weaned at 3 months of age and Wuzhishan pigs were weaned at 1.5 months. The microbial composition of the cecum microbiota of the goat and pig, and the goat rumen were analyzed in months 3 and 12 after cohousing.

Supplementary Figure 2 Estimated completeness and contamination of 3,262 genomes recovered from our datasets. High-quality genomes (completeness ≥ 90%; contamination ≤ 5%) are shown in green, medium-quality genomes (completeness ≥ 70%; contamination ≤ 10%) in purple, and low-quality genomes (completeness ≥ 50%; contamination ≤ 5%) in yellow. Histograms along the *x* and *y* axes show the percentage of genomes at varying levels of completeness and contamination, respectively.

Supplementary Figure 3 Comparison of the read classification rates of published data using the following datasets: a common database consisting of all complete microbial genomes in RefSeq and the GenBank database plus MAGs. The Wilcoxon rank-sum test was used to assess the differences.

Supplementary Figure 4 **(A–C)**: Boxplots are used to summarize the abundance distributions of the abundance of taxa of interest in the gut sections (goat rumen, goat cecum, and pig cecum). The *y*-axis in these panels (except C) indicates the relative abundances, while that in C indicates the abundance ratios between Firmicutes_all and Bacteroidota. Firmicutes_all consists of the following phyla: Firmicutes, Firmicutes_A, Firmicutes_B, and Firmicutes_C. **(D,E)** Microbiota richness and diversity (Shannon index) analyses for each host organ.
