# Supplementary figures and images for: Transmission of the gut microbiome in cohousing goats and pigs
Source: Front Microbiol. 2022 Sep 7;13:948617. doi: 10.3389/fmicb.2022.948617 (PMC9490217; doi:10.3389/fmicb.2022.948617)

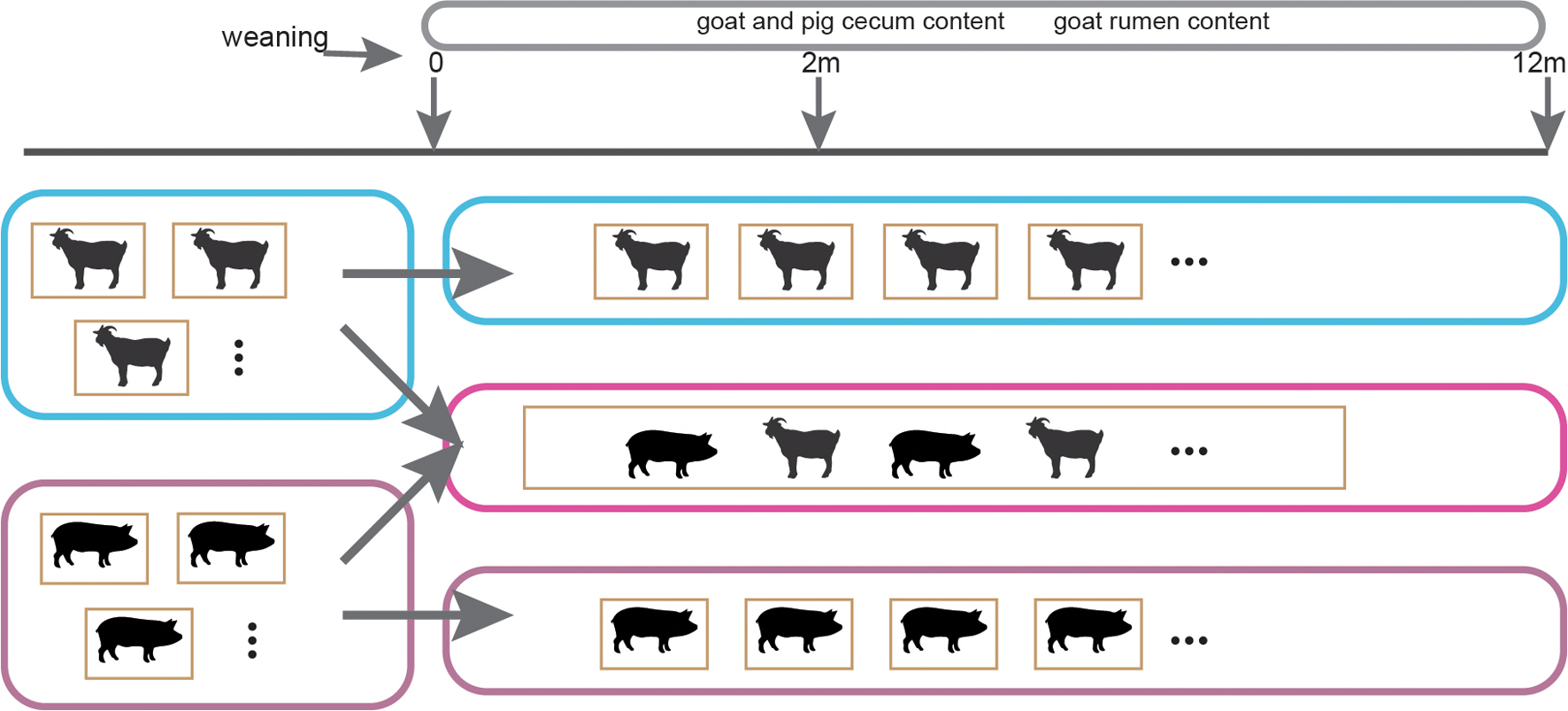

Supplement: Supplementary Figure 1 — Schematic representation of the study design. The experimental design was used to characterize the microbiota composition of the social contact group (co-housed animals; n = 15 Hainan black goats and n = 15 Wuzhishan pigs) and the control group (non-cohoused; n = 15 Hainan black goats and n = 15 Wuzhishan pigs) over time. Hainan black goats were weaned at 3 months of age and Wuzhishan pigs were weaned at 1.5 months. The microbial composition of the cecum microbiota of the goat and pig, and the goat rumen were analyzed in months 3 and 12 after cohousing. [file Image_1.JPEG]

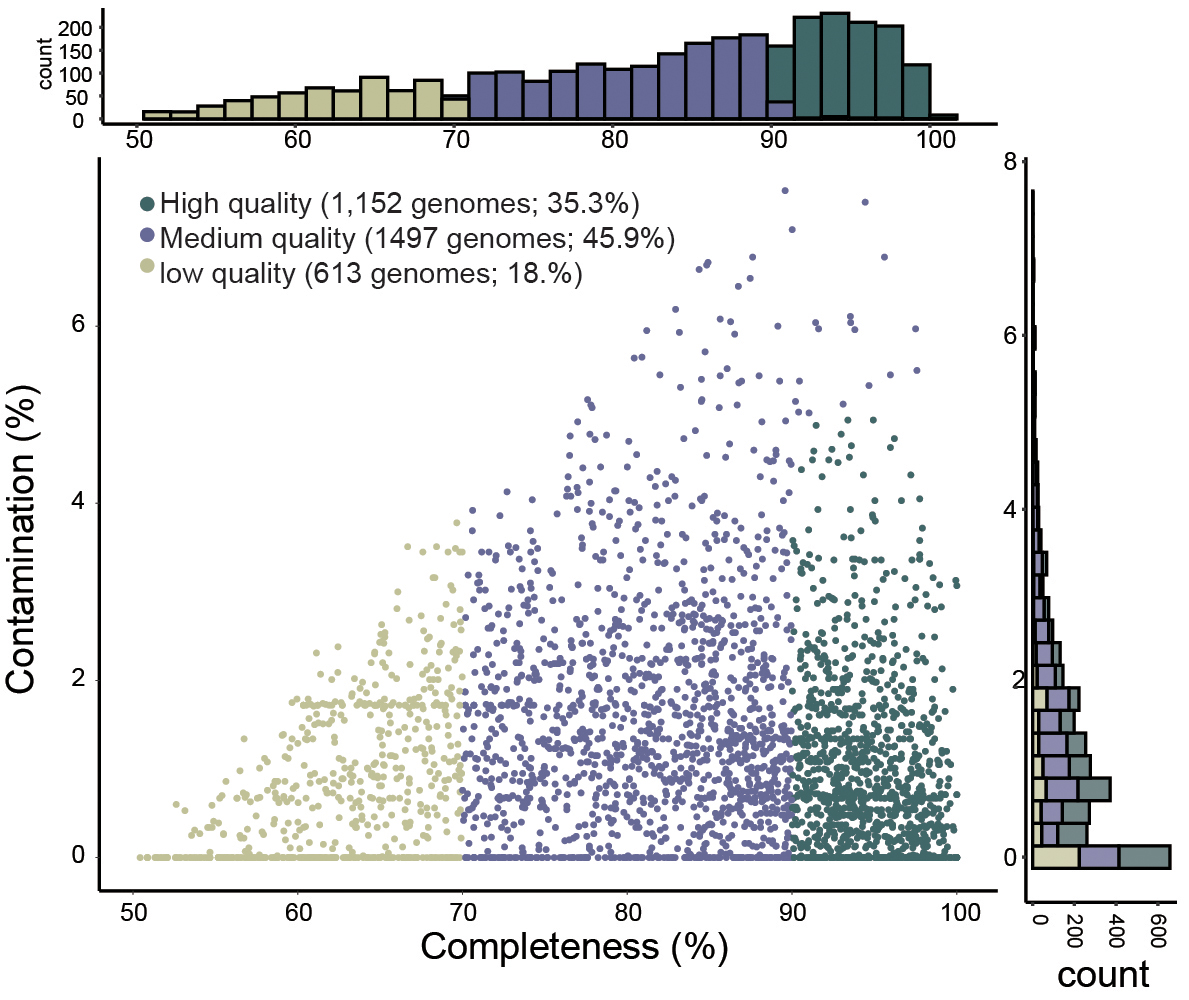

Supplement: Supplementary Figure 2 — Estimated completeness and contamination of 3,262 genomes recovered from our datasets. High-quality genomes (completeness ≥ 90%; contamination ≤ 5%) are shown in green, medium-quality genomes (completeness ≥ 70%; contamination ≤ 10%) in purple, and low-quality genomes (completeness ≥ 50%; contamination ≤ 5%) in yellow. Histograms along the x and y axes show the percentage of genomes at varying levels of completeness and contamination, respectively. [file Image_2.JPEG]

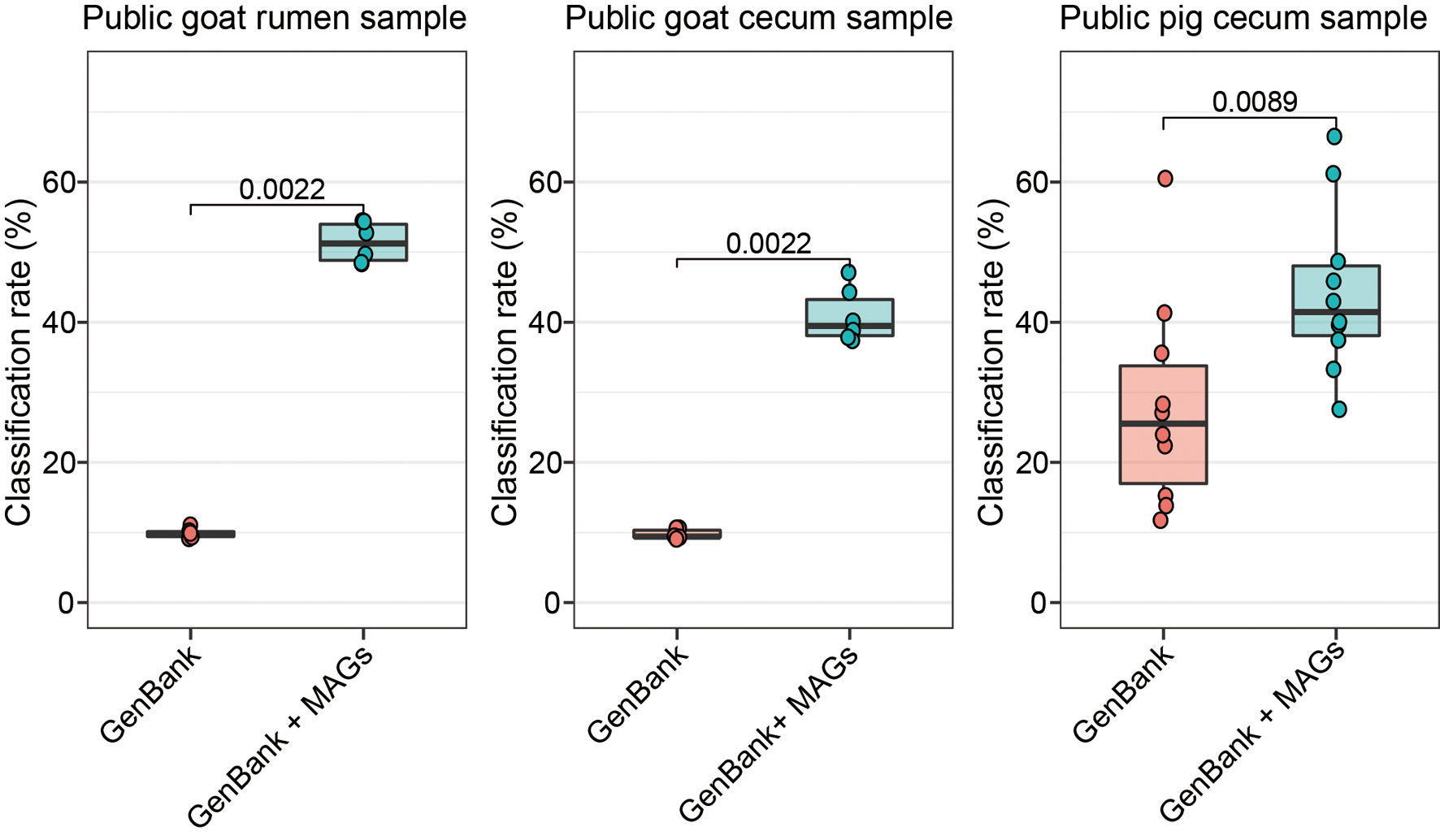

Supplement: Supplementary Figure 3 — Comparison of the read classification rates of published data using the following datasets: a common database consisting of all complete microbial genomes in RefSeq and the GenBank database plus MAGs. The Wilcoxon rank-sum test was used to assess the differences. [file Image_3.JPEG]

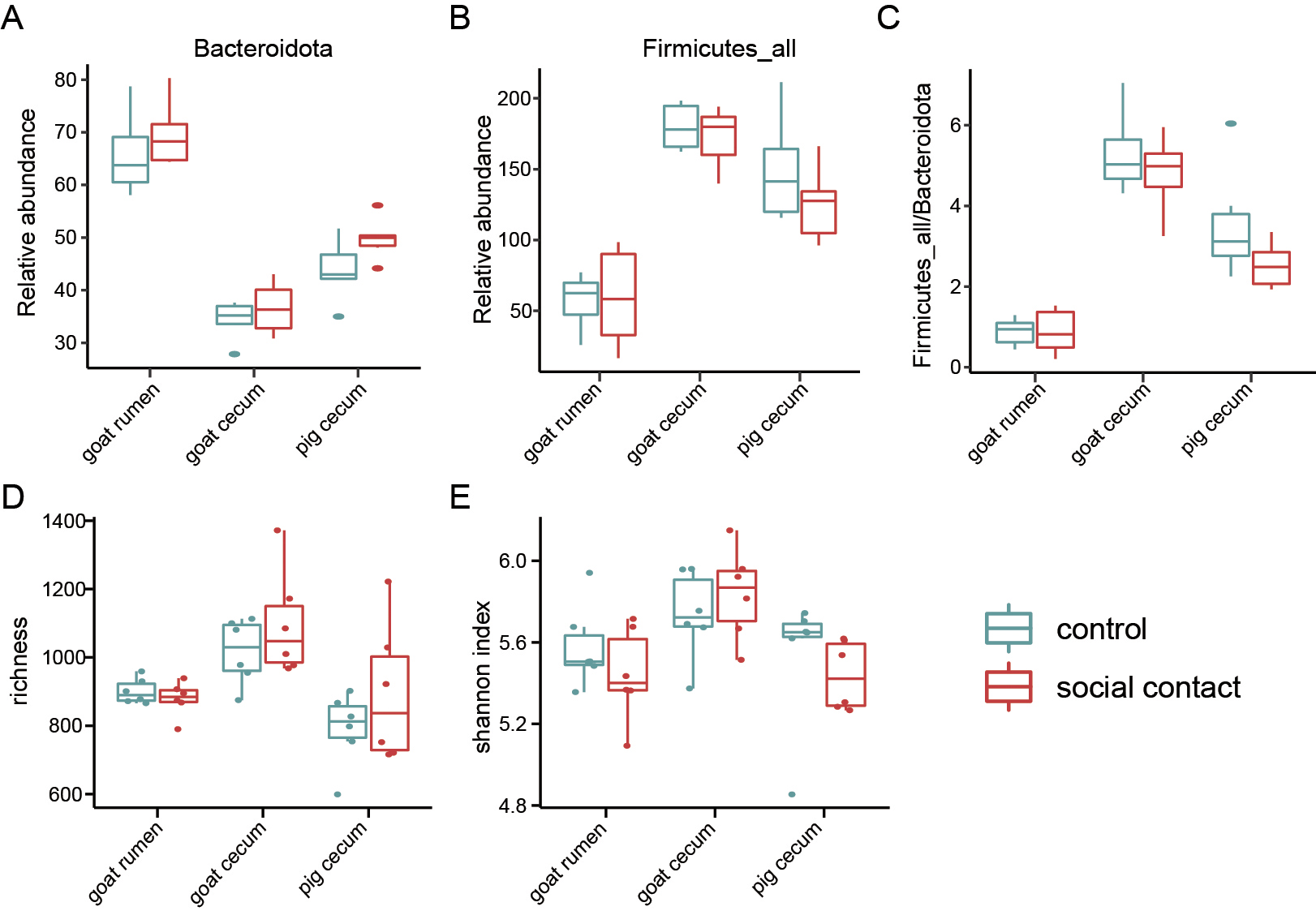

Supplement: Supplementary Figure 4 — (A–C): Boxplots are used to summarize the abundance distributions of the abundance of taxa of interest in the gut sections (goat rumen, goat cecum, and pig cecum). The y-axis in these panels (except C) indicates the relative abundances, while that in C indicates the abundance ratios between Firmicutes_all and Bacteroidota. Firmicutes_all consists of the following phyla: Firmicutes, Firmicutes_A, Firmicutes_B, and Firmicutes_C. (D,E) Microbiota richness and diversity (Shannon index) analyses for each host organ. [file Image_4.JPEG]
